# Positioning zoonotic disease research in forced migration: A systematic literature review of theoretical frameworks and approaches

**DOI:** 10.1371/journal.pone.0254746

**Published:** 2021-07-26

**Authors:** Alex Tasker, Dorien Braam

**Affiliations:** 1 Department of Anthropology, University College London, London, United Kingdom; 2 Disease Dynamics Unit, University of Cambridge, Cambridge, United Kingdom; Jhpiego, UNITED STATES

## Abstract

**Background:**

The emergence and transmission of zoonotic diseases are driven by complex interactions between health, environmental, and socio-political systems. Human movement is considered a significant and increasing factor in these processes, yet forced migration remains an understudied area of zoonotic research–due in part to the complexity of conducting interdisciplinary research in these settings.

**Objectives:**

We conducted a systematic review to identify and analyze theoretical frameworks and approaches used to study linkages between forced migration and zoonotic diseases.

**Methods:**

We searched within eight electronic databases: ProQuest, SCOPUS, Web of Science, PubMed, PLoSOne, Science Direct, JSTOR, and Google Scholar, to identify a) research articles focusing on zoonoses considering forced migrants in their study populations, and b) forced migration literature which engaged with zoonotic disease. Both authors conducted a full-text review, evaluating the quality of literature reviews and primary data using the Critical Appraisal Skills Programme (CASP) model, while theoretical papers were evaluated for quality using a theory synthesis adapted from Bonell et al. (2013). Qualitative data were synthesized thematically according to the method suggested by Noblit and Hare (1988).

**Results:**

Analyses of the 23 included articles showed the increasing use of interdisciplinary frameworks and approaches over time, the majority of which stemmed from political ecology. Approaches such as EcoHealth and One Health were increasingly popular, but were more often linked to program implementation and development than broader contextual research. The majority of research failed to acknowledge the heterogeneity of migrant populations, lacked contextual depth, and insufficient acknowledgments of migrant agency in responding to zoonotic threats.

**Conclusions:**

Addressing the emergence and spread of zoonoses in forced migration contexts requires more careful consideration and use of interdisciplinary research to integrate the contributions of social and natural science approaches. Robust interdisciplinary theoretical frameworks are an important step for better understanding the complex health, environment, and socio-political drivers of zoonotic diseases in forced migration. Lessons can be learned from the application of these approaches in other hard-to-reach or seldom-heard populations.

## Introduction

Zoonotic diseases represent a growing threat to public health [1], with over 60% of newly emerging human pathogens originating in animal species [2]. This burden is felt unequally across populations, with endemic zoonotic diseases accounting for an estimated 20% of human illness and death in low- and middle-income settings (LMIS) [3]. Despite geographic variations in zoonotic disease impacts, recent pandemic outbreaks of Zika, Ebola, and SARS-CoV-2 provide stark reminders of the global importance of interspecies disease transmission. Zoonotic disease emergence is influenced by intersecting political, economic and social factors, operating from global to local levels [4, 5]. Population movements pose significant challenges to the control and prevention of zoonotic disease [6], yet the impact of migration on infectious disease remains poorly understood [7]. Migration, and forced migration in particular, pose unique theoretical [8], methodological [9], and ethical [10] challenges to zoonotic research as forced migrant populations often sit awkwardly between geographies and research disciplines, resulting in the common treatment of forced migrants as homogenized sub-populations in environmental and political studies [11].

Across disciplines, scholars are increasingly recognising the importance of engaging with forced migrants as a distinct population. At the end of 2019, there were an estimated 79.5 million forced migrants, including 20.4 million refugees, 45.7 million internally displaced persons (IDPs), and 4.2 million asylum seekers [12]. LMIS bear the brunt of these displacements; political instability and disasters drive forced migrants to seek refuge within their own, or neighboring countries. Increased international connectivity has resulted in forced migration with profound impacts on global health [13]; factors such as exposure to new ecological environments and endemic diseases [14], limited sanitation [15], deteriorating health services [16], and increased human- and animal stress [17] are thought to drive the emergence and spread of infectious diseases.

The inherent relational complexities of forced migration and zoonotic diseases require a nuanced understanding of social, biological, and ecological factors which have not been adequately studied to date [18]. Traditional epidemiological approaches may struggle to represent the intersections of biological and social processes, creating barriers to accurate representation of the complex factors driving disease emergence, transmission and distribution [19]. The emergence of biosocial theoretical frameworks from epidemiological approaches have provided alternative tools to account for social interactions for understanding these complex interactions, influenced by their epistemological foundations (see, for example, Kingsley and Taylor [20]). As an important emerging field of study, research into zoonoses and forced migration requires a well-developed understanding of the contexts and drivers of forced migration, along with critical reflexivity of theoretical and methodological biases. This systematic review aims to make a significant contribution to the under-researched topic of zoonoses in forced migration by identifying existing theoretical frameworks and approaches used, and critically apprising these to inform future enquiry.

## Materials and methods

### Design

Engaging with the multi-layered and intersecting drivers of zoonotic disease in forced migration contexts presents significant challenges for researchers. Evaluating the quality and utility of existing approaches used to study these complex dynamics is a vital first step in expanding our understanding of this understudied area. Our systematic literature review contributes to this emerging knowledge base by answering the research question: how have theoretical frameworks and approaches been utilized to study the role of forced migration in zoonotic disease dynamics?

**Fig 1:**
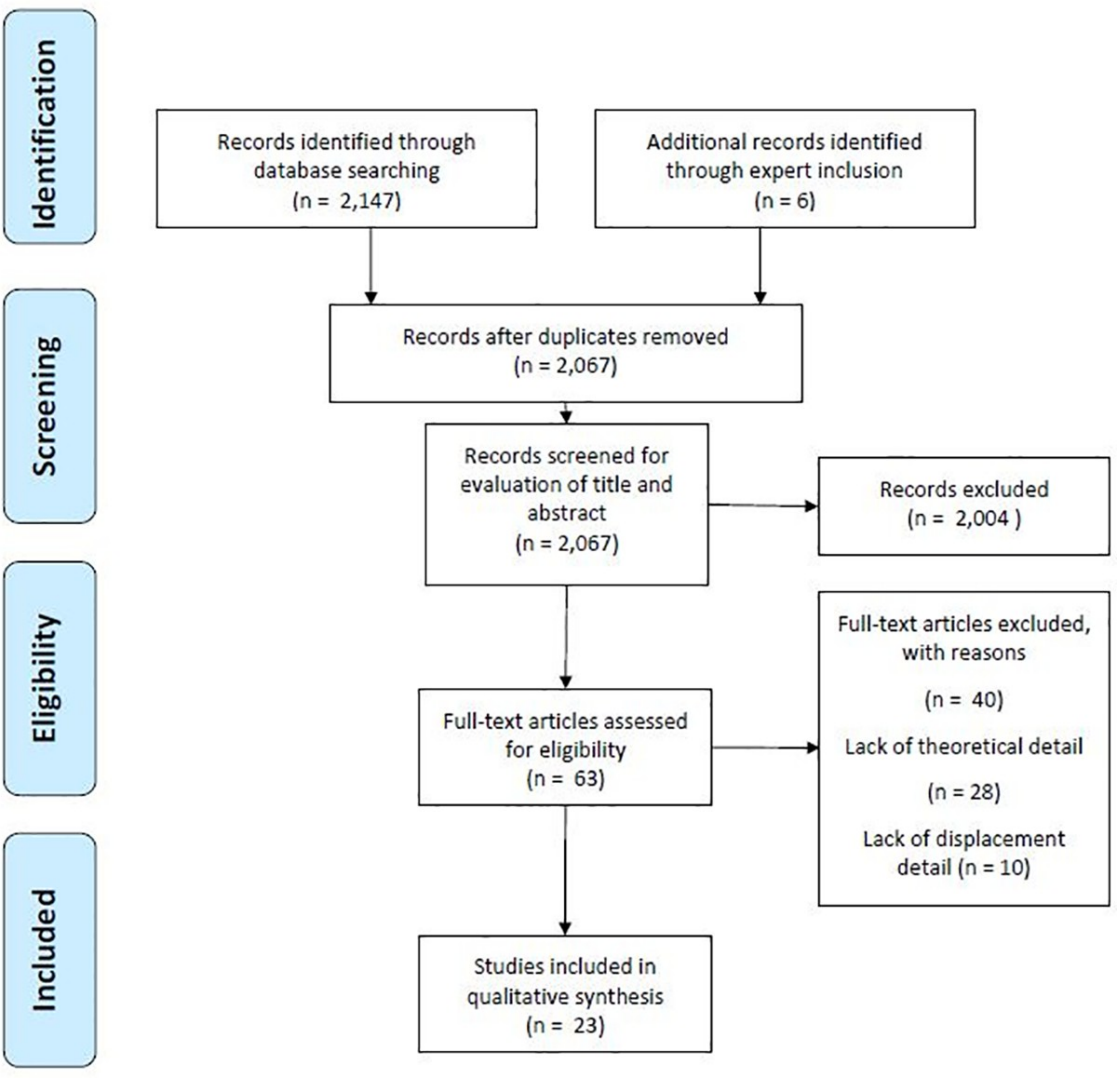
PRISMA flow diagram.

We designed a systematic literature review to identify, collate, and comparatively analyze qualifying literature on zoonoses in forced migration to explore the theoretical and methodological foundations used in these articles. The original protocol for this study is registered with the Open Science Framework, DOI 10.17605/OSF.IO/E9JUF. The study follows the statements on the Preferred Reporting Items for Systematic Review and Meta-Analysis (PRISMA); the PRISMA flow chart is given in Fig 1, the PRISMA statement for this study is provided in S1 Table.

### Search strategy

Eight electronic databases were interrogated in June 2020: ProQuest, SCOPUS, Web of Science, PubMed, PLoSOne, Science Direct, JStor, and Google Scholar, using the search terms zoono* AND ("forced migra*" OR "internal* displace*" OR refugee*). Search terms were developed from wider analyses of zoonotic research, forced migration literature, expert suggestion, and informed by AT and DBs experience. No limit was placed on publication dates.

### Eligibility criteria

Primary inclusion criteria were:

1. Publication of **peer reviewed articles** published **in English after 1975**
2. A **central focus on zoonotic disease**
3. The **specific inclusion** of one or more **forced migrant populations**
4. The **explicit use** of **theoretical frameworks or approaches** at any stage of the study.

### Screening and quality assessment

Citations were downloaded and manually screened for duplicates by the authors (AT and DB). Both authors independently reviewed the complete shortlist of articles using the inclusion criteria above. External experts were consulted individually by AT and DB where either author was uncertain. Disagreements between the authors were resolved by consensus through discussion. The quality of literature reviews and primary data studies were verified using the standardized Critical Appraisal Skills Program (CASP) [21] approach to systematically assess the validity, rigor and contribution of sources to our research question and objective. There remains a lack of a standardized protocol to assess the quality of theory synthesis [22]; we therefore evaluated the quality of theoretical evidence using criteria drawn from a theory synthesis conducted by Bonell et al. [23]. The quality assessment of sources using CASP criteria showed that included literature reviews and primary data were of high quality; the theoretical review revealed a limited number of assumptions, none of which led to disqualification from the study. The results of the combined quality assessment did not result in the exclusion of any studies, but increased the collective rigor of the synthesis [24]. The theoretical quality review is presented in S2 Table, and the CASP quality review in S3 Table.

### Data extraction and synthesis

The synthesis was informed by an inductive qualitative comparative review to identify emergent key themes across the texts in line with the methodologies detailed by Noblit and Hare [25] and Atkins et al. [26]. These themes were then interpreted drawing on wider forced migration and development literatures to explore the applications and limitations of each approach, creating six second order themes and two third-order themes, *population characterization* and *theory and practice*.

## Results

### Search outcomes

The systematic review identified 2,153 articles; removal of duplicates left 2,067 articles. Titles and abstract screening excluded a further 2,004 articles, leaving 63 articles for full-text analysis (see Fig 1). Twenty-three articles met the criteria for inclusion and were included in the comparative analysis, these are given in Table 1 below.

**Table 1:** Publication characteristics and inclusion.

Item	Author, Year	Affiliation	Publication field	Type of study	Location	Displacement	Effect on zoonoses	Theory/ approach
1	Kloos et al., 1988 [27]	Geography	Social Science	Literature review	Ethiopia	Resettlement, refugee, pastoralism, labour migration	Disease distribution	Three-factor disease complex
2	Bouma and Rowland, 1995 [28]	Medicine	Medicine	Prevalence survey	Pakistan	Refugees	Not direct	Sota-mogi model
3	Aiken et al., 1996 [29]	Army	Environment	Review	Bosnia-Herzegovina	Refugees	Importance to military personnel	Environmental health
4	Pedersen, 1996 [30]	Medicine	Social Science	Literature review	Global	Shifting settlement patterns	Interactions human health, development and environmental change	Undefined framework
5	Kalipeni and Oppong, 1998 [31]	Geography	Social Science	Review	Africa	Refugees	Causes of forced migration influence health risks	Political ecology
6	Mayer, 2000 [32]	Geography	Social Science	Review	Global	Refugees, development displaced	Environmental changes	Political ecology
7	Patz et al., 2004 [33]	Environmental health	Environment	Meeting report	Global	Refugees	Environmental and land use change	Systems model approach
8	Schærström, 2009 [34]	Geography	Geography	Review	Global	Refugees	Disease distribution	Disease diffusion
9	Kittinger et al., 2009 [35]	EcoHealth	Ecology and health	Literature review	China	Development displaced	Change in social-ecological dynamics	EcoHealth
10	Confalonieri and Effen, 2011 [36, 55]	Science and Technology	Environmental health	Literature review	Global	Environmental refugees	Human encroachment on ecosystems	EcoHealth
11	Singer, 2014 [37]	Medical Anth	Anthropology	Review	Global	Forced migration	Environmental change and overpopulation	Syndemics
12	Nicole, 2014 [38]	Conservation	Environmental health	Commentary	Uganda	Conservation refugees	Increased poaching	One Health, EcoHealth
13	Machalaba et al., 2015 [39]	Global Health	Global Health	Literature review	Global	Climate change refugees	Expanded suitable habitat pathogens and vectors	One Health, systems approach
14	Nanyingi et al., 2015 [40]	Medicine	Biomedical science	Literature review	Sub-saharan Africa and Arabian peninsula	Refugees	Disease distribution	One Health
15	Whitmee et al., 2015 [41]	Environmental health	Medicine	Commentary	Global	Internal displacement, conflict	Effect environmental change on disease distribution	Planetary health
16	Jones et al., 2017 [42]	Veterinary Medicine	Medicine	Review	Global	Refugees	Ecosocial processes, land-use change	One Health
17	Myers, 2017 [43]	Environmental health	Medicine	Lecture	Global	Conflict and environmental displacement	Human-mediated ecosystem effects	Planetary Health
18	Singer et al., 2017 [44]	Anthropology	Medicine	Review	Global	Refugees	Health inequality	Syndemics, biosocial complex
19	Inci et al., 2018 [45]	Veterinary Medicine	Medicine	Review	Turkey	Refugees	Socioeconomic and environmental changes	One Health
20	Flowra and Azadussaman, 2018 [46]	Public Health	Planetary Health	Commentary	Bangladesh	Refugees	Socioeconomic: overcrowding and hygiene	Planetary Health
21	Abubakar et al., 2018 [47]	Public Health	Public Health	Meeting report	Kenya and Thailand	Refugees	Assumed	One Health
22	Aceng et al., 2020 [48]	Global Health	Public Health	Preparedness plan review	Uganda	Refugees	Social drivers of disease spread	One Health
23	Tomlinson et al., 2020 [49]	Medicine	Global Health	Review	Global	Climate change displacement	Intersections between social, economic, and environmental drivers around mental health	Planetary Health

### Characteristics of included studies

The majority of included publications were theoretical contributions (n = 10), with a limited number of literature reviews (n = 5) and commentary pieces (n = 3). The remaining articles combined meeting reports (n = 2), a lecture (n = 1), prevalence survey (n = 1), and response plan (n = 1).

### Qualitative analysis

We conducted an inductive qualitative comparative analysis of the articles in line with the methodology described by Noblit and Hare [25]. Six second order themes were identified: endemic versus exotic diseases, movement as a vector, homogenisations and generalisations, agency and action, environments and environmental change, health systems and sanitation. These themes were interpreted to identify two second-order themes, population characterization, and theory and practice. The following section reviews the data under these organizing themes.

#### A. Population characterization

Human displacement is driven by a range of complex factors. Within the reviewed literature we identified five typologies of displacement and eight causes, detailed in S4 Table. The majority of articles identified displaced populations as refugees (n = 16), however of these 16, half (n = 8) did not consider the drivers of displacement. Where discussed, conflict (n = 7) and environmental change (n = 5) were the most common drivers; there was a progression towards a more substantiated discussion of conflict and environmental drivers in more recent articles.

Geographically, most publications focused on LMIS populations; 9 from a generalized global view and 11 from a geographically-bounded perspective, including regional (e.g. sub-Saharan or Mediterranean) and country-specific foci (e.g. Turkey or Uganda); details of geographical foci are provided in S5 Table. Despite many studies employing disaster narratives in their framings of specific humanitarian emergencies, there was limited evidence of clustering of locations around discrete disaster events linked to forced migration.

Geographical considerations are central to understanding zoonoses in forced migration. Vulnerabilities of forced migrant populations are rooted in historical and political contexts; drawing on case studies in Africa, Kalipeni and Oppong [31] argue that forced migration should be situated in historical migration events, showing how movements are interlinked with resource scarcity and conflict. The authors note how colonial powers’ common disregard for local contextual understandings has led to the homogenisation of ‘Africa’; the authors cite blind-spots to the uneven distribution of specific refugee populations across the continent as a consequence and symptom of reductionist approaches. Despite these observations, the authors themselves stop short of linking homogenisations to differential individual- and collective disease risks within displaced populations.

The specific contribution of forced migration to zoonotic risks were less well explored. Multiple authors argued that resettlement programs and refugee migration may act as vectors for endemic disease into naive populations, citing increased disease risk through environmental and socioeconomic change such as poverty and health inequality [42, 46, 47]. The earliest work included in this review [27] suggested these factors be addressed though improved co-ordination of development programs; while subsequent work by Mayer [32] developed the need to disentangle disease risks in forced migration by improving understanding of biological drivers of disease dynamics. The author argued this was particularly true of dense and unhygienic settlements–forced migrants fell into this category. Despite acknowledging a need for greater depth of understanding, the majority of authors stopped short of engaging with the wider complexities of human migratory movements. Meanwhile, forced migration studies (as opposed to those specifically dealing with zoonoses) recognize how drivers of migration may have significant implications for health [50]. For example, McMichael [51] distinguishes between voluntary and forced migration, noting that “*well-managed migration could provide an adaptive response that reduces the adverse health impacts of climate change*” [51, p.549].

#### B. Theory and practice

The second theme identified tensions between research framed as more abstract theoretical enquiry, and more empirical, holistic approaches informed by a combination of theoretical precedent and contextual understandings. Adherence to structured theory during research remains a contested subject across disciplines, with views ranging from ‘there is nothing so practical as a good theory’ [52, p.18] to suggestions that the use of theory in research may inhibit intellectual engagements [53]. Theoretical foundations, and associated conceptual frameworks, are commonly used to construct and validate both question and evidence, and as such are highly political processes that shape the outcomes of scholarship. We identified the use of fifteen discrete theoretical frameworks across the 23 studies, conducted by researchers from 11 disciplines and sub-disciplines. The first publication to explicitly employ a theoretical framework was published in 1988, with articles steadily increasing over time up to 3 per year in 2015, 2017, and 2018. We observed a subjective movement from the use of social science perspectives such as political ecology (n = 2) and systems theory (n = 2) from the late 1990s-early 2000s towards more cross-disciplinary engagement employing Planetary Health (n = 3) and One Health (n = 7) lenses in the last six years. Our review included four studies (structured reviews (n = 3) and reports (n = 1)) which gave little consideration to nuanced discussions around theory. The following section discusses each in turn; firstly more abstract theoretical studies, followed by more contextually-rooted work. A brief summary of models and frameworks recorded in this study, arranged alphabetically, is included in Table 2 below. Where we considered models closely related for the purpose of zoonotic disease research, we combined them here for ease of presentation.

**Table 2:** Summary of frameworks and approaches.

**EcoHealth and ecological models of health**
The EcoHealth approach attempts to understand connections between human, animal, and ecosystem health, rooted in ideas of environmental sustainability and socioeconomic development. EcoHealth explicitly acknowledges the influence of political, economic, and cultural systems on diverse ecological landscapes, describing how these factors drive the nature, concentration, and relationships influencing disease in human populations [54]. EcoHealth research draws on complex systems thinking, integrating community engagement, gender, and equity analyses [55], using transdisciplinary action-research to overcome the conceptual and practical limitations of more traditional approaches [56]. EcoHealth’s centring of ecological and socio-economic determinants of disease transmission have been criticised for neglecting economic and policy aspects [57]; despite these arguments, EcoHealth research continues to grow in popularity.
**Ecosocial theory and processes**
Ecosocial theory aims to improve understandings of epidemiological risk factors, and to develop hypotheses around the social dynamics of health and disease [58]. Ideas of embodiment are central to ecosocial theory, centring connections between personal, institutional, and communal bodies in driving patterns of health, disease, and well-being [59], influenced by power, property, production and consumption, acting on individual experiences, gender, age and health behaviours, influencing individual biology and health outcomes [60]. Ecosocial research is often notable in the use of contextual and multilevel approaches to analyse individual- and community-level data in ecological and social contexts [58]. The multidisciplinary nature of ecosocial research provides a powerful tool for gathering diverse perspectives, but this complexity may limit its wider use by epidemiologists.
**Environmental health**.
Environmental health offers one of the broadest conceptualisations of human-environmental connections, but often lacks a clear and focused framework for research. The language of environmental health has been widely adopted by the World Health Organization (WHO), who commonly reference environmental health when advocating for healthier environments to prevent global disease burdens. Environmental health normally considers a range of factors from the built environment such as healthy and safe cities and workplaces, adequate water, sanitation and hygiene, protection from chemicals and radiation, and sound agricultural practices, to the natural environment around healthy climate, air and preserved nature.
**One Health**
One Health approaches seek to improve interdisciplinary collaborations between veterinary and human medicine, while encouraging engagement of ecological and environmental sciences [61]. One Health conceptualizes health as an outcome of social-ecological system dynamics, an approach that necessarily requires cross-disciplinary cooperation and collaboration from human and animal health professionals to assess social and environmental drivers of health [56]. One Health frameworks developed by international organisations successfully increased international coordination and cooperation during the H1N1 influenza pandemic in 2009 [62]. Despite proving its usefulness for addressing social domains of zoonoses, One Health has received criticisms for inadequately developing and articulating the approaches philosophical and methodological foundations behind apporaches [63]. These critiques have led to increasing attention on the gaps between veterinary and human health knowledge silos, and the perceived exclusion of alternative disciplines [64].
**Planetary Health**
Planetary Health approaches seek to conceptualise the complex relationships between human health and place across multiple scales and domains [65]. Many planetary health studies focus on the effect of environmental disruptions driving health impacts within local and global communities, including atmospheric, oceanic, and territorial changes [43]. Many of these arguments centre on resource scarcity as a driver of environmental exploitation; these acts effect health through multiple pathways, including impacts on nutrition, communicable and non-communicable diseases, human movement, and conflict [43]. Planetary health approaches provide a useful tool for conceptualising links between macro- and micro-level effects, but have attracted criticisms for a lack of clarity around the mechanisms through which these effects are generated and felt; researchers continue to systems-based methodologies to address these concerns [66].
**Political ecology and Ostrom’s economic theory**
Political ecological theories and frameworks are well established tools for researching human-environmental relationships [67]. The complex interplay of social, political, economic, and ecological factors have very real impacts on the health and wellbeing of populations, with authors such as Pedersen [30] establishing links between disease models and economic growth, conflict, nationalism, and development. For the exploration of zoonoses, political ecology often supports other approaches such as One Health by providing multi-scale, contextual analyses of underlying historical, political, and socio-economic vulnerabilities to disease. Notably, political ecological approaches enable close consideration of individual and population-level coping mechanisms and outbreak responses [68]. Political and economic understandings of resource use underpin many of these interpretations; recent critiques of economic growth and human development theories have problematized aspects of political ecology dealing with human-environmental relationships [69]. Ostrom, a notable political economist, challenged mainstream thinking around community-governed ’commons’ [70]. Rather than leading to overuse and depletion through shared ownership and social arrangements, Ostrom’s theorem suggested that communities with low income but high levels of social capital exchange successfully managed these sites, leading to improvements health outcomes [71].
**Sota-mogi model**
Host-vector relationships are central to the spread of zoonotic disease. Researchers have modelled transmission between actors to explain disease dynamics, and to develop public health interventions. Malarial zooprophylaxis, the mitigation of mosquito bites through domestic animals, was evaluated in the 1940s [72] and led to further work modelling the effect on mosquito population dynamics of multiple bloodmeal hosts. The first mathematical description of these dynamics was provided by Sota and Mogi [73] who concluded that domestic animals influenced mosquito population density, and therefore were a key factor in bites frequency and malaria endemicity in human populations, limiting the predictive and explanatory power of the model around livestock movement in driving zoonotic disease emergence and spread.
**Spatial diffusion theory & disease diffusion**
Spatial diffusion approaches seek to map the drivers and progression of disease through transmission events. Schærström [34] suggests these events may be conceptualised either as the co-incidence of cases in space, or as sites of intersecting risk factors. Approaches that seek to localise cases often fixate on ultimate health outcomes, whereas consideration of co-incident drivers rebalances this thinking towards causes of disease. This differentiation becomes more important when considering disease transmission and spread; fundamentally, public health interventions can seek to inhibit individual-to-individual transmission, or alter environmental drivers promoting the spread of disease. Drawing on epidemiological studies of influenza, hepatitis, and AIDS incubation and spread, researchers have stressed the importance of understanding time and space when thinking about diffusion. The value of considering disease diffusion through a spatial-temporal framework is in part that it roots epidemiological thinking in wider landscapes that account for social movement and ecological change.
**Syndemics & biosocial complex approach**
Singer et al. [54] developed syndemic theory to provide a framework with which to explore relationships between social conditions and disease concentrations, typified by work on HIV/AIDS distributions amongst food insecure populations in Southern Africa [54]. Syndemic approaches examine how and why specific groups and individuals are more vulnerable to specific diseases as a function of existing ill health, social, and environmental marginalisation [44]. Syndemic theory centres ideas of social inequality and injustice as drivers of disease; these approaches suggest disease clustering is a factor of structural oppression, driven by poverty, stigmatization, stress and structural violence (Singer, 2017).
**Systems and systems model approaches**
The emergence and transmission of zoonotic diseases are relational acts, which at the collective level form complex systems. System-based heuristics are commonly employed to research complexity; frameworks such as ‘systems thinking’ and ‘systems models’ have underpinned research across many domains for decades [74]. Despite widespread use, the core concepts and approaches of systems research are often poorly defined [75]. For infectious disease research, systems thinking and systems models offer multiple benefits [76], including shaping public health interventions [77]. When employed in a rigorous manner, systems-based perspectives enable researchers and policymakers to conceptualise a wide range of variables that may drive health outcomes. Successful applications of systems thinking include the ability to account for behavioural variation, policy inputs, economic data, and health impacts driving tobacco use [78], and the effects of land use and climatic variation on public health [33].
**Three factor disease complex**
In the early 1950s, Medical Geographies were proposed as a lens through which to examine health. [79]. Typologies were used to explain diseases outcomes resulting from parasitic infections, characterised as ‘two-, three-, or four-factor complexes’ [79, p.24]; numeric ally identified as the number of organisms perceived as necessary to propagate disease. A range of case studies were presented to support these complexes, many of which are found in migrant populations including Cholera (‘two-factor’, humans and *V*. *cholerae*), Malaria (‘three-factor’, human, *Plasmodium*, and *Anopheles*), and Tsutsugamishi disease (‘four-factor’, Trombiculinae, rodent, *Rickettsia orientalis*, and human). These complexes highlight the importance of human-animal transfer for zoonotic disease, however the approach lacks engagement with the wider social and ecological drivers of disease that contribute to the proximity of these organisms, and the context of transfer.

*Disciplines and theoretical frameworks*. Kalipeni and Oppong [31] explicitly employed a political ecological framework to suggest how disease dynamics frameworks should move beyond separate consideration of biophysical and environmental factors in forced migration. The authors highlighted how political and social forces underpin refugee crises, leading the authors to conceptualise unsanitary refugee camps as spaces that could lead to the emergence of zoonotic diseases. This framing links political and ecological drivers of refugee movement to negative health outcomes for forced migrants, through which the authors explicitly advocated for high-level responses to protect global health against zoonotic disease. These responses, they argue, must be understood through a nuanced multilevel historical contextualization of intersections between environmental and societal factors, including human agency in environmental changes.

Themes of environmental change can be found in many of the included studies. Writing two years later, Mayer [32] again used a political ecological approach to explore disease ecologies of emerging zoonotic infections, this time rooted in a biomedical framework which incorporated the unintended consequences of human actions from individual to global levels. Pedersen [30] developed the political aspects of environmental change further, using a human rights-based lens to argue that ecosystems are overburdened by the ’pursuit of unlimited economic growth’ [30, p.745]. This view placed the responsibility of zoonotic mitigation on high-level policy makers to directly engage with disease emergence. Pedersen argues in their study that natural resource conflict and development synergistically drive forced migration, which results in the burdens of disease being borne by the displaced; an insightful argument that resonates with many contemporary investigations of links between migration and health inequalities [80]. Despite a promising theme of enquiry, the author does not develop this position further to suggest how specific impacts of disease dynamics are driven, or felt by forced migrant populations.

Parallel to macro-political landscapes of inequalities and health, many studies in this review structured their research so as to present empirical case studies which explored the influence of national and international health systems on disease pathways. Spatial diffusion theory used by Bouma and Rowland [28] characterized Pakistani refugee camps as disease transmission nodes, driven by overcrowding and unsanitary living conditions, but limited attention was given to the specific mechanisms by which this may occur. An alternative exploration of disease diffusion was given by Schærström [34] who emphasized the individual and environmental challenges posed by these sites, once again the mechanics of these factors was left uncovered. In contrast, Singer [37] explicitly highlighted anthropogenic factors in mass population migration which may contribute to zoonoses through the development of syndemic theory. In their earlier paper, Singer suggested how microbial changes are linked to social and environmental conditions which in turn underpin zoonotic threats; these themes are revisited in their later paper through consideration of social justice, disease prevalence, and health inequalities driven by poverty, stress, and structural violence [44]. We suggest that this later article represents a step forward in bridging multiple dimensions of zoonotic disease emergence in forced migration, most notably through the recognition of heterogeneity within specific study sites at global and local levels.

As noted earlier, social justice was discussed by both Pedersen [30] and Singer [37]. In their report on the Anthropocene to The Rockefeller Foundation and Lancet Commission, Whitmee et al. [41] continue this debate using a Planetary Health framework, calling for the integration of ’multisectoral actors’ [41, p.2006] to transform health systems. A Planetary Health framework is again employed by Myers [43] to explore case studies of Ebola, HIV, and schistosomiasis through which they highlight links between human-ecosystem relationships and disease exposure risks. Myers argues that environmental changes are driven by social processes and population characteristics; this position is echoed by Flowra and Asaduzzaman [46] who consider climatic drivers of microbes and infection as linked to social changes. In parallel to the growth in non-forced migration literature exploring intersections between social and global environmental change, many of the later papers in this review recognized climatic change as a key factor in understanding the emergence and transmission of zoonoses. Myers [43] suggested environmental changes drive negative health outcomes for forced migrants through processes of malnutrition, infectious disease, and trauma. In parallel, Tomlinson et al. [49] detail the role of global environmental change as a potential driver of forced migration. Both of these studies frame their discussion of zoonotic disease outbreaks as a feature of increased overcrowding, deforestation and wildlife encroachment; both studies once again stop short of fully exploring the complexity and variability of the social and biological mechanisms leading to these outcomes.

*Approaches for research and implementation*. When evaluating zoonoses in forced migration, it is important to understand the often opaque relationship between theoretical and applied approaches used by researchers. For example, political ecology is often characterized as a theoretical approach, whereas frameworks including One Health and EcoHealth resist easy definition, variously employed as theory, methodology, and a policy devices [81]–tools for both research and implementation. One Health approaches in this study were most commonly described as frameworks for either a) exploring eco-social processes driving infectious disease emergence and spread in human and animal populations [42], or b) tools for program development [47]. In this review, One Health approaches were specifically applied to the behavior and movement of reservoir species for zoonotic disease pathogens [39, 40], and the exploration of zoonotic disease risks from poverty and poor hygiene [82]. These studies varied in location, population, and depth, ranging from granular explorations of respiratory infections in Kenyan and Thai refugee settlements [47] and Ebola in Congolese refugees in Ugandan camps [48], to more macro-level evaluations of Rift Valley Fever [40] and Nipah [39]. In this last example, the total engagement with One Health was as an aim for future work. Regardless of the topic and depth, no study which employed One Health acknowledged the role of historical, political and socio-economic drivers in their evaluations.

EcoHealth approaches in forced migration research emerged almost in parallel with One Health. EcoHealth shares much common ground with One Health, providing systems-based ways of thinking around zoonoses, disease emergence, and pandemic threats [56]. EcoHealth is often cited as contributing a more nuanced understanding of ecosystem drivers of disease than One Health [61], though it is unhelpful to consider either as a superior tool for holistic evaluation [83]. In the studies considered for this review, EcoHealth approaches were most commonly used to explore land use change and disease; using the framework, Patz et al. [33] discuss refugee movements as vectors for Tuberculosis and Hepatitis B, Confalonieri and Aparicio Effen [36] suggest Nipah links to land use change and Lyme disease through increased contact with host diversity, and Kittinger et al. [35] explore interactions with snail hosts of *Schistosomiasis*.

## Discussion

Despite covering over three decades of work, the articles considered in the review consistently display a number of shortcomings. Most articles treated forced migrants as exemplar populations, proxies for exposure to specific and isolated risks, without acknowledgments or consideration of the inherent complexity and diversity of these populations. Multiple studies reviewed here chose to link human mobility to endemic and non-endemic disease exposure, reducing forced migrants to vectors for the movement of non-endemic disease into study populations. We argue that the reality for forced migrants is significantly more complex. Classifying populations as forced migrants for instance overlooks the fact that many within these communities may have been settled for many years [27]; indeed evidence suggests that disease patterns are similar between long-established forced migrant populations and host populations living in comparable living conditions [28]. From Africa [84], Asia [85], to Europe [86] and beyond, researchers are increasingly arguing for robust consideration of endogenous heterogeneity of migrant populations, and those at the margins of state control. For example both newly migrated, and long-term residents are situated within widely differentiated social networks [87]. These relationships offer diverse and differentiated resources that can shape zoonoses transmission, detection, and treatment. This complexity is compounded by the highly contextual nature of many endemic zoonotic diseases, operating within distinct ecological ranges. We argue that this renders generalisations solely on the basis of national borders unhelpful, especially when considered through the lens of long-term informal cross-border movements. From our previous research, we support the idea that communities labeled as *migratory* may often have more in common with settled indigenous groups in border zones than the wider national population. This underlines the importance of understanding political and social context when framing zoonotic disease risks in migrant populations, in particular the unique institutional landscape in which many refugees exist [88].

In addition to internal diversity and contextual detail, all studies considered in this review failed to consider migrant-led responses to zoonotic disease. Many articles cited poor sanitation and ineffective health systems, yet forced migrants have in the past adapted their surroundings through local water supply innovations [89], and developed internal networks for health provision [90]. In the last decade, multiple studies have detailed the endogenous creativity of migrant populations [91], to the point that refugee self-reliance has on occasion been co-opted as a development tool, arguably to the detriment of the communities in question [92, 93]. Despite external political influence, without acknowledgment of migrants’ individual agency, and the dynamic nature of forced migrants’ relationships with zoonotic disease, we suggest that research is likely to fail to accurately capture the complexity of these sites of disease emergence and transmission.

Our review does give cause for hope. The subjective shift from the political ecological approaches of the 1990s and early 2000s, towards a more Public Health-based understanding in the last decade suggests a movement towards a more holistic, interdisciplinary treatment of complexity. These approaches are arguably better positioned to map the nonlinear nature of zoonoses which are driven by a variety of social, political, historical, economic, environmental, and biological factors. Advances in One Health and EcoHealth approaches have arguably outpaced wider understandings of the role of human migration on infectious disease, but we are optimistic that increasing attention offers the chance to bring these domains together. Despite this optimism, the centering of Public Health in forced migration zoonotic research should prompt critical reflection, informed by current debates on neocolonial aspects of international aid and development programming. Public Health has the ability to generate highly techno- and ethnocentric narratives which, rather than engaging local communities in healthcare, can serve to obfuscate indigenous knowledge systems and further disadvantage the most vulnerable. Considerations of social justice can be found across articles include in the review [30, 32, 49]; as research moves to engage with the inherent complexity of zoonotic research in forced migration, we suggest that actors involved in forced migration, including national- and international NGOs, governments, and activist groups, should explore how these approaches may reflect local understandings and systems of power, rather than providing a façade for the business-as-usual of international assistance.

## Limitations of the review

There are several limitations to this review. Only English language articles were included, limiting our engagement with indigenous scholarship. Secondly, it is possible that ethnographic studies exist that engage with zoonotic diseases using locally-defined terms which would not have been detected by the search protocol. This review only considered peer reviewed articles, making publication biases likely and selecting against internal reports from development organizations. Given the focus of this review was primarily theoretical, it reports and evaluations which did not explicitly employ theoretical or analytical frameworks will have been excluded.

## Conclusions

The results of this review indicate that research on zoonotic diseases in forced migration increasingly employs interdisciplinary approaches to consider holistically social, environmental, and biological complexity. These approaches acknowledge linkages between human, animal, and environmental health through the integration of disciplines and participation of local stakeholders and interlocutors. Many of these approaches build on political ecological approaches to address macro-level disease risks, but often overlook the complexity and diversity of forced migrant populations. Studies continue to characterize movement as a major determinant for disease and health outcomes, despite a lack of evidence for doing so. Disease vulnerabilities depend on a multitude of internal and external factors which must be better researched and contextualized; further work is required to build theoretical frameworks able to address the societal and biological processes of forced migration across time and space.

## Supporting information

S1 Fig PRISMA flow diagram. Fig 1: PRISMA flow diagram.
(TIF)

S1 Table PRISMA statement. Table 1: PRISMA statement.
(DOCX)

S2 Table Theoretical quality review. Table 2: Theoretical quality review.
(DOCX)

S3 Table CASP quality review. Theoretical quality review.
(DOCX)

S4 Table Typologies and causes of displacement. Typologies and causes of displacement.
(DOCX)

S5 Table Geographical focus and distribution of studies. Geographical focus and distribution of studies.
(DOCX)
