# Supplementary figures and images for: Positioning zoonotic disease research in forced migration: A systematic literature review of theoretical frameworks and approaches
Source: PLoS One. 2021 Jul 26;16(7):e0254746. doi: 10.1371/journal.pone.0254746 (PMC8312951; doi:10.1371/journal.pone.0254746)

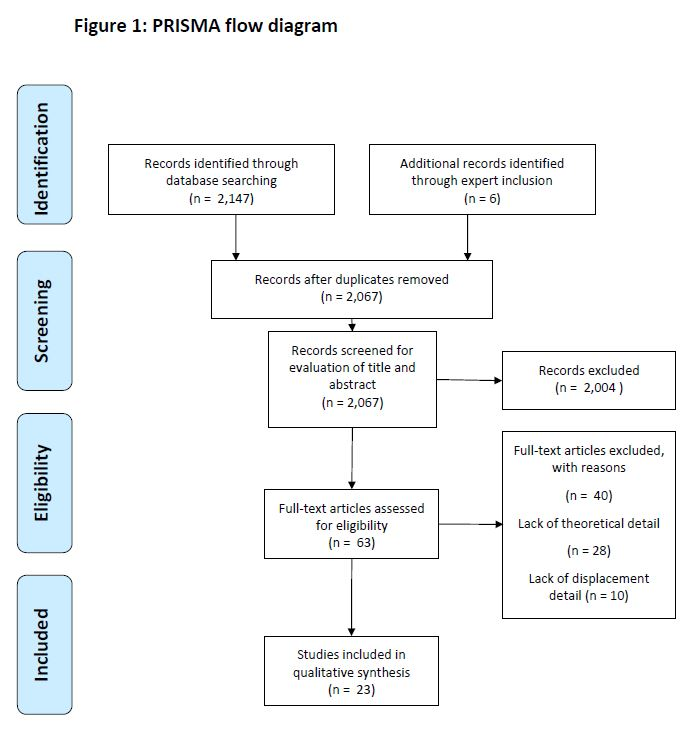

Supplement: S1 Fig — Fig 1: PRISMA flow diagram. (TIF) [file pone.0254746.s001.tif]
